# Toe Walking as the Initial Symptom of a Spinocerebellar Ataxia 13 in a Patient Presenting with a Mutation in the KCNC3 Gene

**DOI:** 10.1055/s-0041-1736483

**Published:** 2021-10-19

**Authors:** David Pomarino, Johanna Ronja Thren, Anneke Thren, Kevin Rostasy, Jan Schoenfeldt

**Affiliations:** 1 PTZ Pomarino, Hamburg, Germany; 2 Department of Anthropology, Durham University, Durham, United Kingdom; 3 Diakoniekrankenhaus Annastift, Hannover, Germany; 4 University of Witten/Herdecke, Witten, Germany; 5 Institute for Pediatric Neurology, Hamburg, Germany

**Keywords:** SCA 13, spinocerebellar ataxia 13, toe walking, idiopathic toe walking, habitual toe walking, genetic testing toe walking, pes cavus

## Abstract

This article at hand described a 4-year-old child patient who initially presented with the symptoms of toe walking. As part of the diagnostic process, the patient was genetically tested to find the cause of the gait anomaly. The genetic test found a mutation in the KCNC3 gene. The variant c.1268G > A; p.Arg423. His was found in a heterozygotic state. This variant is frequently described as a cause for spinocerebellar ataxia type 13 (SCA13) in the literature. Apart from toe walking as the most pronounced symptom, the patient displayed an instable gait with frequent falls and delayed speech development. The genetic test to determine the cause of the gait anomaly successfully diagnosed the patient with a previously undiscovered SCA13 and subsequently enabled the recommendation of personalized further treatment.

## Introduction

The term “toe walking in children” describes a characteristic gait anomaly, where patients walk on their forefoot for most of the time. Toe walking can have different neurological and orthopaedic causes and is often associated with further illnesses. 1 This makes both the professional treatment of patients and a thorough diagnostic process very important. If the anamnesis finds no alternative clinical cause of the gait anomaly, the patient's gait is often described as “idiopathic toe walking”. This terminology is misleading, as recent publications suggest that previously undiscovered genetic mutations lead to the symptoms of toe walking when there is no apparent alternative clinical cause. 2

For the first time, this article described the case of a mutation in the KCNC3 gene in relation to toe walking. The full clinical report and the results of the genetic test are described in the subsequent paragraphs.

## Case Description

The male patient was sent to our specialist practice for gait anomalies at the age of 4 years, following a recommendation by their pediatrician. The patient had previously been treated by a physiotherapist as part of an integrated service at their kindergarten. The anamnesis found that the patient was spontaneously born in the 40th week of pregnancy following an infection with B-streptococcus. The birth weight was 3680 g and the patient's height at the time of birth was 49 cm. The patient developed pneumonia in infancy and was treated with antibiotics and mask-assisted breathing in hospital; following treatment the patient was released in generally good health. The patient took his first independent steps when he was 11 months old. From gait onset, the patient walked with an instable gait and fell frequently; after walking for 30 minutes the patient was extremely exhausted. No signs of muscle weakness or epilepsy were found. The toe walking intensified substantially as the patient got older. There were no known chronic or neurological health conditions or gait anomalies in the family. The patient's parents are not consanguine.

The neurological-motoscopic report, following an examination at the age of 4 years, found an atactic gait pattern, with bilateral toe walking, as well as a broadened forefoot, bilateral drop foot, and pes cavus. The patient reported no pain resulting from the abnormal gait. Furthermore, the patient exhibited a hyper lordosis of the lumbar spine with an angle of 40 degrees. The mobility of the upper ankle joint for dorsal extension/plantar flexion was 5–0–50 degrees with a straight knee and 10–0–50 with the patient's knee bent. The examination also found a straight hip and spine position. The proprioceptive reflexes were successfully triggered bilaterally, reflex zones were not broadened, and the Babinski sign was negative. No disruption was found in the cranial nerve region and there was no dysarthria or disruption of the ocular movement. An magnetic resonance imaging found a pronounced narrowing of the cerebellar Gyri and of the corresponding marked Sulci as signs of a cerebellar atrophy. No further structural abnormalities were found.

Genetic testing found a mutation in the KCNC3 gene of the heterozygotic variant c.1268G > A; p.Arg423His. This genetic change was found to be the likely cause of the present SCA13-ataxia. 3 This variant has been linked to a significant loss of activity in the tension-dependent potassium channel in previous studies. SCA13-ataxia is hereditary via the autosomal dominant channel and displays a characteristically slow progression with a very varied age of onset ranging from infancy to late adulthood. 4 5 6 7 The clinical variant was not found in either of the patient's parents.

## Therapy

The therapeutic treatment process included a consultation with a pediatric neurologist. The treatment of the gait anomaly was conducted using lower leg night splints and personalized pyramid insoles to achieve an improved gait and reduce the intensity of toe walking in the patient. Following treatment, the parents reported that the patient would rarely walk on the forefoot and would display toe walking predominantly when going barefoot. The patient can walk unassisted at the age of 5 years but struggles with a coordinative weakness and frequent falls. The maximum walking distance is significantly reduced and the patient experiences frequent trembling because of the underlying disease. The use of a wheelchair was recommended to help manage this and avoid the need to carry the patient on longer walks. The use of a therapy bike is also being considered. It is recommended that the night splints continue to be worn and cupped insoles have been prescribed to be worn during the day. Additionally, it was recommended that physiotherapeutic treatment should be continued.

## Conclusion

This article provided the first-hand description of a patient, who displays toe walking as a main symptom of a SCA13-ataxia as a result of a mutation in the KCNC3 gene. Consequentially, the patient exhibits toe walking with a genetic cause. The diagnosis was secured following genetic testing, where the identification of the ataxia was relevant for subsequent treatment and contributed to the correct treatment choice for the patient.
